# Autonomous exoskeleton reduces metabolic cost of human walking during load carriage

**DOI:** 10.1186/1743-0003-11-80

**Published:** 2014-05-09

**Authors:** Luke M Mooney, Elliott J Rouse, Hugh M Herr

**Affiliations:** 1 Mechanical Engineering Department, Massachusetts Institute of Technology, Cambridge, MA, USA; 2 MIT Media Lab, Massachusetts Institute of Technology, Cambridge, MA, USA; 3 Harvard-MIT Division of Health Sciences and Technology, Massachusetts Institute of Technology, Cambridge, MA, USA

## Abstract

**Background:**

Many soldiers are expected to carry heavy loads over extended distances, often resulting in physical and mental fatigue. In this study, the design and testing of an autonomous leg exoskeleton is presented. The aim of the device is to reduce the energetic cost of loaded walking. In addition, we present the Augmentation Factor, a general framework of exoskeletal performance that unifies our results with the varying abilities of previously developed exoskeletons.

**Methods:**

We developed an autonomous battery powered exoskeleton that is capable of providing substantial levels of positive mechanical power to the ankle during the push-off region of stance phase. We measured the metabolic energy consumption of seven subjects walking on a level treadmill at 1.5 m/s, while wearing a 23 kg vest.

**Results:**

During the push-off portion of the stance phase, the exoskeleton applied positive mechanical power with an average across the gait cycle equal to 23 ± 2 W (11.5 W per ankle). Use of the autonomous leg exoskeleton significantly reduced the metabolic cost of walking by 36 ± 12 W, which was an improvement of 8 ± 3% (*p* = 0.025) relative to the control condition of not wearing the exoskeleton.

**Conclusions:**

In the design of leg exoskeletons, the results of this study highlight the importance of minimizing exoskeletal power dissipation and added limb mass, while providing substantial positive power during the walking gait cycle.

## Background

The ability to carry substantial loads is required by many professions, including many that may experience cognitive deficits associated with the extreme physical demands. For example, soldiers are often expected to carry loads between 20–35 kg at speeds of 1.5-1.75 m/s for over 10 km in a single march [1,2]. Wheeled vehicles, however, are excellent at reducing the effort of carrying substantial loads, but terrain and space restrictions often limit the practicality of a vehicle and require the versatility of legged locomotion. Exoskeletons have the potential to reduce the energetic cost of carrying such loads while maintaining the flexibility of legged locomotion. To this end, one of the first leg exoskeletons that posited to augment human legged locomotion was patented in the late 19^th^ century [3]. Since that time, interest in developing exoskeletons to augment strength and endurance has increased substantially, driven by the accelerating pace of innovation in several mechanical and computer-related disciplines [4-6]. Reducing the metabolic energy (*i.e.* energy from food) consumed by the human body during legged locomotion is the goal of many exoskeletal technologies [7,8]. To our knowledge, no autonomous leg exoskeleton system has been demonstrated to reduce the metabolic demand for either walking, loaded walking or running. In this work, “autonomous” describes a device that is self-contained and has all necessary components on-board (e.g. power supply, controller, and actuators, with no tether to external systems).

Researchers have attempted to reduce the metabolic burden of load carriage by developing both passive and active exoskeletons. Walsh et al. presented a device that used springs in parallel with the ankle and hip joints along with a variable damper at the knee [9]. A preliminary study showed that the device transferred 80% of a 36 kg payload to the ground, but a 10% metabolic increase was observed while wearing the exoskeleton. Kazerooni and Steger developed a powerful lower extremity exoskeleton for load carriage with actuated hip, knee and ankle joints [4]. The device allowed a user to walk at 1.3 m/s while carrying a 34 kg payload along with the 36 kg exoskeleton, but a metabolic improvement was not shown. Both of these devices distributed the payload weight through the structure of the exoskeletons, thus reducing the weight borne by the human user. The device presented by Walsh et al. achieved this by outputting high knee damping during the early stance phase of walking, while Kazerooni and Steger’s device provided active power at the joints to support the load. However, the added mass of these sophisticated exoskeletons may have limited their abilities to improve metabolism.

Exoskeletons have also been developed to assist with unloaded locomotion. To achieve this goal a class of passive exoskeletons have been designed. Donelan et al. developed a device that uses an electromagnetic generator to harvest energy from the knee in a biomimetic fashion [10,11]. Harvesting energy during periods of negative power at the knee was less metabolically detrimental than continuous harvesting, but a metabolic increase of over 20% was still observed. Recently, van Dijk et al. presented an exoskeleton that has a spring spanning the ankle, knee and hip joints [12]. The average energy expenditure of walking increased by over 30% while wearing the device. Although passive exoskeletons have not reduced the metabolic cost of walking in healthy individuals, they have been shown to augment the energetics of hopping [13,14]. Adding passive elements in parallel with human joints are able to assist the muscles during periods of spring like behavior. Such work is encouraging, but the extension to exoskeletons designed for walking is unclear.

Many factors have hindered the development of an autonomous performance-enhancing exoskeleton including substantial added mass, limited mechanical power and tethered energy supplies. Autonomous devices capable of providing biologically equivalent levels of joint mechanical power necessary for locomotion have been investigated, but these devices were cumbersome and heavy [7,15,16]. Adding mass to the lower limbs requires additional metabolic power, and the effects are amplified as the mass is moved distally or further away from the hip [17]. In an effort to reduce the weight of the worn exoskeleton, researchers have developed passive and quasi-passive exoskeletons. Without an active actuator, these devices are not able to provide levels of positive power that overcome the negative metabolic effects of added device mass [9,10,12].

Researchers have reduced weight and provided substantial positive power by tethering exoskeletons to an energy supply not worn by the user [18-22]. The device of Malcolm et al. was able to provide a 6% metabolic improvement during walking using pneumatic artificial muscles. This device required a tether to an air supply and extensive valving control network, thus distancing it from an autonomous solution. Despite the tethered nature of this devices, the study shows that it is possible to reduce the metabolic cost of walking by assisting the ankle with a lightweight device capable of providing positive mechanical power.

The purpose of this study is to present the design and testing of an autonomous leg exoskeleton capable of reducing the metabolic cost of walking with load. The intent of this research is to develop a technology that can assist individuals who must carry loads for extended periods of time, such as soldiers. Our hypothesis is that a leg exoskeleton capable of providing substantial levels of positive mechanical power with minimal added distal mass can provide such a metabolic benefit. In the evaluation of this hypothesis, we augment the ankle joint because it is responsible for over 50% of the average positive mechanical power during loaded walking [23]. We test the metabolic effect of the ankle exoskeleton during walking with load carriage, and present the Augmentation Factor, a general framework of exoskeletal performance that unifies our results with the varying abilities of previously developed exoskeletons.

## Methods

### Device design

**Figure 1:**
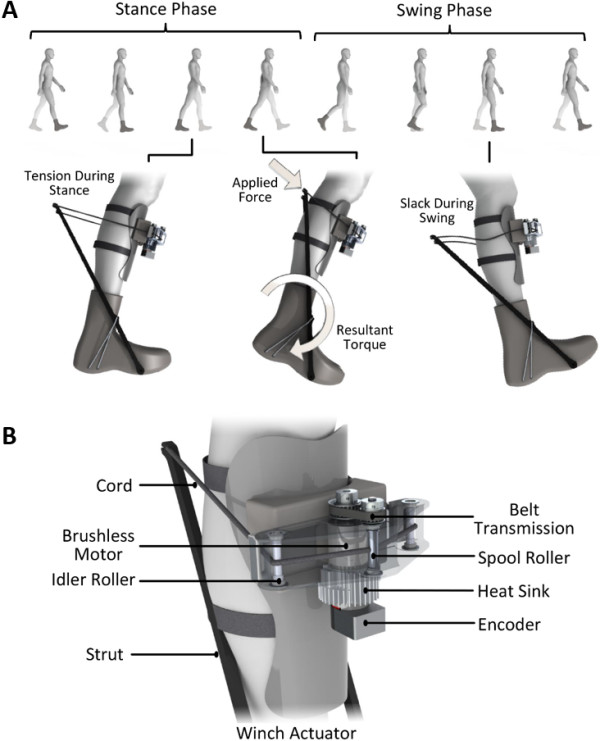
**Autonomous leg exoskeleton. (A)** The autonomous exoskeleton applies torque about the human ankle joint during walking, adding positive mechanical power to the wearer during the push-off portion of stance phase. During the swing phase, the device applies negligible forces on the wearer by allowing small amounts of slack into the cord. The mechanism consists of a winch actuator and fiberglass struts that directly apply a resultant torque about the ankle. **(B)** The winch actuator provides a torque on the ankle by winding the cord around the spool. As the cord is tightened, a force is applied to the struts on either side of the leg. The winch actuator’s brushless motor applies the torque to the ankle joint through a transmission that consists of the belt transmission stage in series with the geometric transmission stage comprising spool, idler roller and strut.

The bilateral exoskeleton was designed to provide assistance to the ankles during walking and was comprised of three main assemblies: a pair of fiberglass struts attached to each boot, unidirectional actuators mounted on the anterior shank segments, and a battery and control package worn on the waist (Figure 1). Each boot had a medial and lateral fiberglass strut pinned to the front of the boot, one at the medial and one at the lateral aspect of the metatarsophalangeal joints. Each strut was coupled to the heel of the boot via a lightweight inextensible cord. The fiberglass struts were an extension of the ankle-foot complex; when an anterior force was applied to the proximal end of the strut it was converted into a torque about the human ankle joint. The struts acted as a moment arm (≈300 mm from the center of rotation of the ankle joint) for the winch actuator to apply the plantar-flexion assistive torque about the ankle joint. There are four forces that acted on the strut: the force of the winch cord on the proximal end of the strut, the reaction force of the heel cord, the reaction force of the pin attaching the distal end of the strut to the boot, and the force of the ground on the distal end of the strut. The location of the heel cord attachment to the strut was chosen in such a way that the reaction force at the distal end of the strut was mostly in the vertical direction, which is provided by the ground and not the pin itself. This reduces the necessary size and weight of the pin. The exoskeleton used a custom winch actuator powered by a brushless DC (BLDC) motor. The 200 W BLDC motor (model: 305015, Maxon Motor, Sachseln, CH) actuated an 8 mm diameter spool through a belt transmission with a 13:8 speed reduction. The spool wrapped a 1 mm diameter ultra-high molecular weight polyethylene cord (Dyneema, Stanley, NC) attached to the proximal end of the fiberglass struts. The effective transmission ratio between the BLDC motor and ankle joint was approximately 120:1. The geometric transmission, comprised a spool, idler roller and strut, eliminated the need for a traditional mechanical transmission, reducing weight and complexity of the device.

The actuation systems of the exoskeleton were powered and controlled by the batteries and motor controllers worn around the waist. Sensory information was provided to the controller; each boot contained instrumented insoles that detected heel and toe contact with the ground (model: FSW, B&L Engineering, Santa Ana, CA). The angular position of the BLDC motors were measured with 500 count quadrature incremental optical encoders (model: HEDL 5540, Maxon Motor, Sachseln, CH). Each winch actuator had a corresponding BLDC motor controller (model: SBL1360, Roboteq, Scottsdale, AZ). The motor control loop iterated at 1000 Hz. The motors, sensors and controllers were all powered by two 24 V lithium-polymer batteries, with a capacity of 2.5 Ah each. The total mass of the system was 4.0 kg, with 1.7 kg worn on the waist and 2.3 kg worn on the legs; a list of major component masses and locations are shown in Table 1.

**Table 1:** Exoskeleton component mass distribution

**Part**	**Mass (g)**	**Location**
Footswitch	103 (x2)	Foot
Strut assembly	273 (x2)	Foot & Shank
Winch actuator	749 (x2)	Shank
BLDC Controller	316 (x2)	Waist
Batteries	400 (x2)	Waist
Waist pack	281	Waist
**Total**	**3963**	

Components that have both a right and left version are denoted with (x2), and the shown mass is only for one exoskeletal leg.

A biomechanically-inspired control strategy was implemented to assist the user during the push-off portion of stance phase and become transparent to the user during swing phase. During stance phase, the controller used a linear model of the motor to produce open loop torque profiles. The details of the linear motor model are further discussed in the *Actuator Testing Protocol* subsection. Upon heel contact, the actuator behaved like a soft virtual spring. The equilibrium position of the soft virtual spring was set to the peak plantar-flexion angle achieved just after heel strike. During the controlled dorsiflexion region of stance phase, the winch actuator applied a slight plantar-flexion torque that linearly increased with the extension of the winch cord. This soft virtual spring maintained tension in the cord and allowed for a gradual increase in applied torque. The step period of each subject was measured while not wearing the exoskeleton. The controller was then set to initiate push-off assistance at 43% of the subject’s personal gait cycle time [22]. During the push-off portion of stance phase, the actuator exerted a large plantar-flexion torque. Over 50 ms, the exoskeleton increased the applied plantar-flexion torque by approximately 120 Nm. Subsequently, the applied plantar-flexion torque linearly decreased with plantar-flexion angle. Once the actuator reached 0.2 radians of ankle plantar flexion, the controller entered a zero applied torque mode (swing phase). This mode did not impede additional plantar flexion by the biological ankle due to the nature of the unidirectional actuator. During swing phase the exoskeleton provided slack in the drive cord to allow the user to freely dorsiflex the biological ankle. To prevent such interference, the motor proceeded to the maximum angle reached during controlled dorsiflexion of the previous cycle. Stance phase was reinitiated when the controller detected heel contact from the insole and any slack in the cord was eliminated with a low applied torque that was imperceptible to the user.

### Actuator testing protocol

In order to reduce distal mass and complexity, the exoskeleton does not have an integrated force sensor; therefore, a linear model of the BLDC motor was used to predict the mean mechanical power provided by the exoskeleton. The current and torque of the BLDC motor were estimated by

(1a) $$I=\frac{V-k_{v}\omega }{R}$$

(1b) $$\tau =k_{t}I-J\overset{˙}{\omega }$$

where *I* is the motor current, *V* is the applied voltage, *k*_*v*_ is the motor voltage constant, *R* is the terminal resistance, *ω* and $\overset{˙}{\omega }$ are the motor’s velocity and acceleration, *τ* is the estimated motor torque, *k*_*t*_ is the torque constant, and *J* is the rotor inertia. The motor’s angle, as measured by the encoder, was numerically differentiated to get the motor velocity and then differentiated again to get the motor acceleration. The velocity differentiation was calculated by a single finite difference. The acceleration was not calculated for real-time control due to the low rotor inertia. However, the acceleration was calculated by the central-difference method during post-processing to estimate the mean power supplied by the exoskeleton.

The mechanical power applied by the exoskeleton, *p*, was calculated as the product of the estimated motor power and experimentally determined mechanical power efficiencies for regions of positive and negative power, *η*^*+*^ and *η*^*-*^. The mechanical power provided by the motor was calculated as the product of *τ* and *ω*. The positive mechanical motor power was then scaled by *η*^*+*^, and the negative mechanical motor power was scaled by *η*^*-*^, to estimate the mechanical power applied by the exoskeleton,

(2) $$p=\left\{\begin{array}{cc} \eta^{+}\tau \omega & \tau \omega >0\\ \eta^{-}\tau \omega & \tau \omega <0 \end{array}\right)$$

The transfer efficiencies are defined as the ratio of the mean measured mechanical power output of the actuator and the mean predicted actuator power:

(3a) $$\eta^{+}=\frac{p_{\text{exp}}^{+}}{p^{+}}$$

(3b) $$\eta^{-}=\frac{p_{\text{exp}}^{-}}{p^{-}}$$

where $p_{\text{exp}}^{+}$ and $p_{\text{exp}}^{-}$ are the experimentally measured average positive and negative mechanical powers, and *p*^*+*^ and *p*^*-*^ are the estimated average positive and negative mechanical powers provided by the exoskeleton . The transfer efficiency of the actuator was experimentally determined with an inline force sensor and acquisition equipment (model: LRF350, Futek Advanced Sensor Technology, Inc., Irvine, CA). The force sensor had a max rated force of 890 N, and a sampling rate of 250 Hz with 12 bit resolution. The winch actuator was used to pull the inline force sensor and stiff elastic band attached to the ground. The winch cord linear velocity was derived from the motor rotational velocity, transmission and spool diameter. The measured power, the product of the cord linear velocity and measured force, was compared to the estimated power, the product of the motor velocity and estimated torque.

### Walking protocol

The metabolic effect of the exoskeleton was tested on seven male subjects (84 ± 6 kg; 181 ± 5 cm; 23 ± 4 years old; mean ± standard deviation) walking on a treadmill at 1.5 m/s with a 23 kg weighted vest. These conditions were chosen because they are consistent with typical loads and speeds experienced by soldiers [1,2]. All subjects were healthy and exhibited no gait abnormalities. Only subjects with a foot size between 11 and 12.5 were considered to ensure proper boot fit. This study was approved by the MIT Committee on the Use of Humans as Experimental Subjects. Consent was obtained from experimental participants after the nature and possible consequences of the exoskeletal studies were explained. The experimental protocol involved three walking trials and one standing trial, all performed while wearing a portable pulmonary gas exchange measurement instrument (model: K4b^2^, COSMED, Rome, IT). To account for natural variation in metabolism, the control condition of no exoskeleton was tested before and after the exoskeleton condition. The subjects first walked for 10 minutes with no device. After the subjects donned the exoskeleton, they walked with the active device until they reported being comfortable with the assistance, typically less than 5 minutes. The subjects then walked for 20 minutes with the exoskeleton (Additional file 1), in order to allow for human-machine adaptation [24]. The subjects then walked for another 10 minutes with no device. Finally, after the last no device trial, subjects stood for 5 minutes with the weighted vest and no exoskeleton in order to obtain the resting metabolic rate.

Metabolic rate was calculated from oxygen and carbon dioxide exchange rates measured by the portable pulmonary gas exchange measurement unit. The average flow rates of the last two minutes of each trial were converted into metabolic power using the equation developed by Brockway et al. [25]. The metabolic rate of standing was subtracted from the metabolic rate of walking trials in order to obtain the net metabolic cost of walking. The net metabolic rates measured from the two control trials were averaged and compared to the net metabolic rate of the exoskeleton trial [20,24]. A paired *t*-test was used to test for metabolic improvement, with the level of significance set at 0.05.

The mechanical and electrical powers of the exoskeleton were wirelessly recoded via Bluetooth at a sampling rate of 100 Hz. The mechanical power applied by the winch actuators were estimated through the linear motor model and experimentally measured transfer efficiency discussed in the previous section. The electrical power was also recorded on a subset of four subjects by measuring the battery voltage and current during a period of walking with the exoskeleton.

### Augmentation factor

A simple model to estimate the metabolic demand of an exoskeleton design would be a valuable tool for designers, but there are many factors which may affect exoskeletal performance. Sawicki and Ferris presented the performance index as a metric of measuring the relationship between applied positive exoskeleton mechanical power and change in metabolic power [19]. A higher performance index suggests that the exoskeleton is able to more efficiently transfer mechanical power into metabolic power. The efficient transformation of mechanical power into metabolic power is important for autonomous systems that must carry their own energy supply. The generality of the performance index enables the comparison of various exoskeletal designs, but it is limited to developed and experimentally tested devices. The performance index cannot make a prediction about the metabolic performance of an untested exoskeleton.

**Figure 2:**
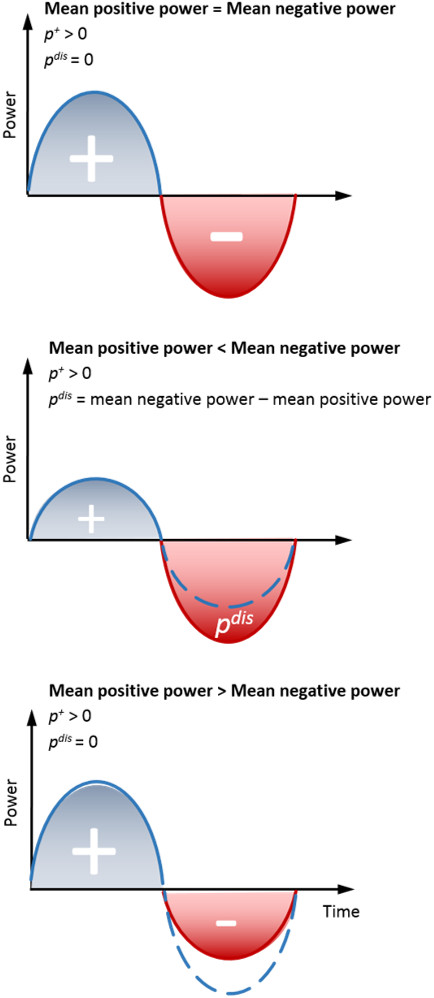
**Calculation of negative net-power dissipation term, *p***^***dis***^**. **Various power profiles are shown with the corresponding positive and dissipative powers. Note that when mean positive power is greater than the mean negative power, the dissipative term is zero.

As a resolution to this difficulty, we developed a general model called the Augmentation Factor (AF) to predict the metabolic impact caused by a worn exoskeleton at moderate walking speeds. Commonly, devices attempt to improve metabolic rate by either providing positive mechanical power to joints during phases of positive muscle-tendon power, providing negative mechanical power to joints during phases of negative muscle-tendon power, or a combination thereof [9,10,20,22]. The AF defined as

(4) $$\mathrm{AF}=\frac{p^{+}+p^{\text{dis}}}{\eta }-\overset{4}{\underset{i=1}{\sum }}\beta_{i}m_{i}$$

estimates the change in metabolic power caused by a worn exoskeleton. The AF predicts the metabolic improvement of a device in Watts. When computing the AF for a device, a positive AF suggests that use of an exoskeleton will result in a metabolic improvement whereas a negative AF indicates that an exoskeleton would increase walking metabolism. The AF balances those mechanical exoskeletal properties that cause a metabolic improvement with those properties that cause a metabolic detriment; the AF balances the mean positive mechanical power (*p*^*+*^) supplied by an exoskeleton with the net mechanical power dissipation (*p*^*dis*^) and added device mass on each limb segment (*m*_*i*_). The AF increases with *p*^*+*^ since adding positive mechanical power to the body reduces positive muscle-tendon work. In distinction, the AF decreases if an exoskeleton removes more mechanical energy from the body than it supplies, denoted by a negative net-power dissipation (*p*^*dis*^). The musculoskeletal complex utilizes elastic elements to efficiently store and release energy during level ground walking [8,26,27]. Negative net-power removed by an exoskeleton, *p*^*dis*^, cannot be stored elastically and reused by the body, resulting in additional positive muscle-tendon work and an increased metabolic rate. A graphical representation showing the relationship between *p*^*+*^ and *p*^*dis*^ is shown (Figure 2). The muscle-tendon efficiency, *η*, is used in (4) to convert exoskeletal mechanical power to metabolic power. This value was obtained by taking the mean of efficiency values empirically determined in previously published exoskeleton studies, *η* = 0.41 mechanical Watts for every metabolic Watt [20,22]. As such, 41 W of positive mechanical power supplied by an exoskeleton replaces 100 W of metabolic power. The empirically obtained efficiency is consistent with previous studies investigating the positive muscle fascicle work efficiency of 0.25 [28-30], which is a lower efficiency limit for joint positive power since energy stored elastically in tendons and ligaments and released during concentric muscle-tendon contractions have a positive impact on such an efficiency [19,20]. Finally, the AF also accounts for the metabolic burden due to additional leg mass by the sum of the products of the device location factors (*β*_*i*_) and the exoskeletal component masses (*m*_*i*_). The included exoskeletal masses are those worn on the foot, shank, thigh and waist (enumerated by *i* in equation 4). The device location factors (*β*_*i*_) are 14.8, 5.6, 5.6, and 3.3 W/kg from ankle to waist, respectively [17]. Browning et al. provided linear regression equations for each segment that relate added mass to net metabolic rate normalized by body mass. The device location factors were obtained by taking the product of the linear regression slopes and the average subject mass studied by Browning et al.

## Results

### Actuator calibration

The calibration tests were performed with a sinusoidal motor position profile, over a range of frequencies (1–4 Hz) with an amplitude of 150 mm. The corresponding force amplitudes ranged from 0 to 350 N. These values were chosen because they are similar to the actuator range of motion and force experienced during walking. The calibration tests demonstrated a positive power transfer efficiency (*η*^*+*^) of 0.68 ± 0.01 (mean ± standard error) and a negative power transfer efficiency (*η*^*-*^) of 0.77 ± 0.01, resulting in an average root mean square error of 10 W over the range of tested frequencies and amplitudes. These efficiencies along with (1a), (1b) and (2) were used to estimate the power applied by the exoskeleton during human trials.

### Metabolic and mechanical power

**Figure 3:**
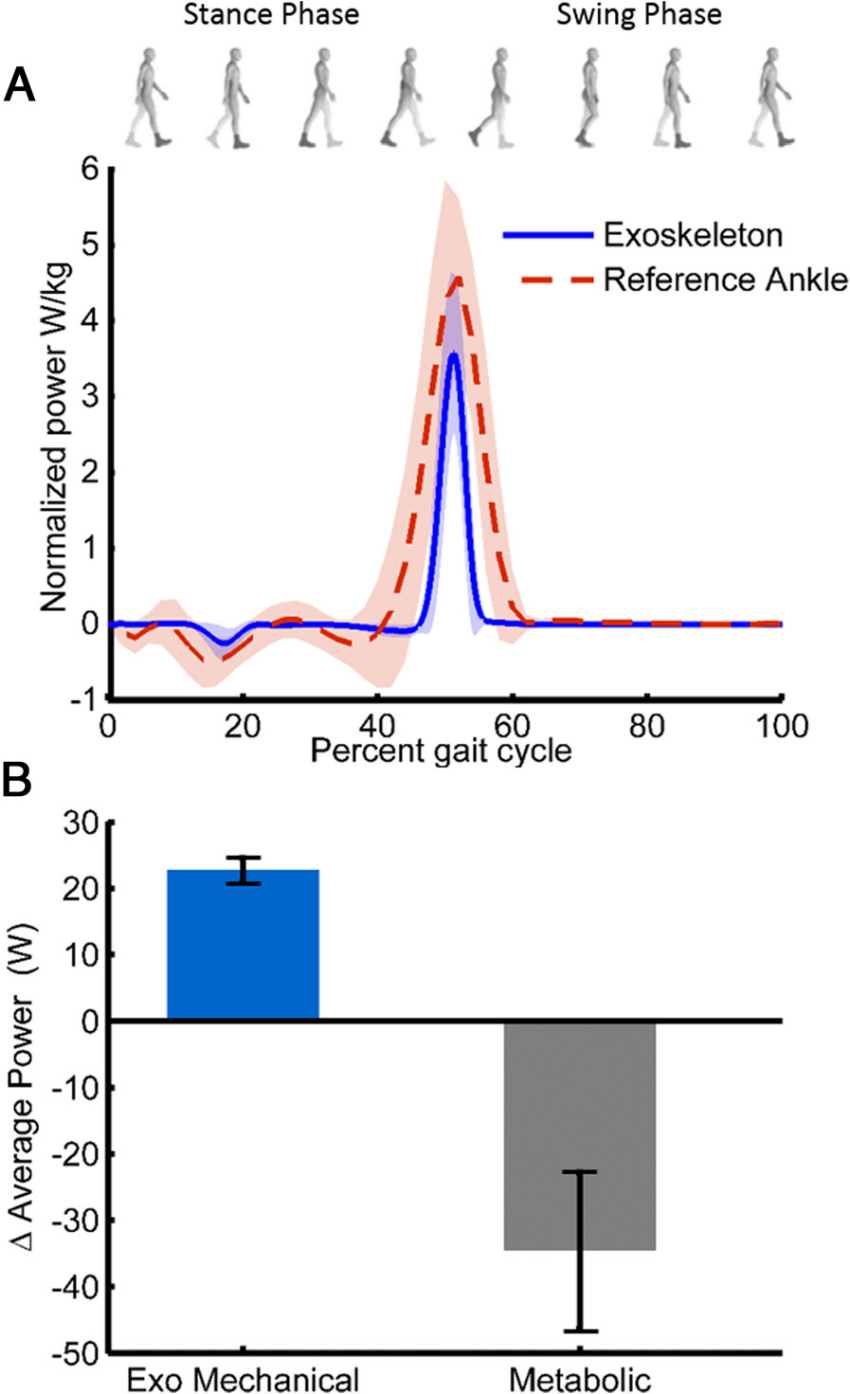
**Mechanical and metabolic results of wearing the autonomous exoskeleton. (A)** Inter-subject mean exoskeletal ankle power provided by only the exoskeleton is shown (blue) throughout a single gait cycle, while carrying load. Power is normalized by body mass with standard deviation shown in translucent. For comparison, the mechanical power provided by only the biological ankle joint is shown (dashed red) in the case of fast walking without a load or exoskeleton, acquired from a reference dataset [33]. The normalized maximum mechanical power produced by the ankle while walking with a 20 kg load has been shown to increase to over 6 W/kg [23]. **(B)** Inter-subject mean change in mechanical and metabolic power is shown when using the exoskeleton is compared to not using the exoskeleton, with error bars denoting standard error. The increase in exoskeletal mechanical power demonstrates how much positive mechanical power is provided to the wearer by the exoskeleton. The decrease in metabolic power demonstrates the reduction in the rate of metabolic energy consumed while wearing the exoskeleton.

The exoskeleton significantly reduced the metabolic power required to walk at 1.5 m/s with a 23 kg load. Standing with the 23 kg load required 1.85 ± 0.11 W/kg and walking without the exoskeleton required 6.98 ± 0.24 W/kg, agreeing with previous work [31,32]. The average metabolic cost of walking with the exoskeleton was 6.56 ± 0.29 W/kg. Use of the autonomous leg exoskeleton significantly reduced the metabolic cost of walking by 36 ± 12 W, which was an improvement of 8 ± 3% (*p* = 0.025) relative to the control condition (Figure 3). The exoskeleton reduced the metabolic cost for six of the seven subjects. During walking, the exoskeleton applied a mean positive mechanical power of 23 ± 2 W (11.5 W per ankle) to the wearer during the push-off portion of stance phase (Figure 3). The metabolic and mechanical power results for each subject are shown in Table 2.

**Table 2:** Metabolic power, mechanical power and augmentation for seven subjects

		**No Device**	**Exoskeleton**		
**Subj.**	**Mass**	**Metabolic**	**Metabolic**	**Augmentation**	**Avg. mech. pow.**
	**(kg)**	**(W)**	**(W)**	**(%)**	**(W)**
1	82	397	338	14.6	23.4
2	75	408	378	7.4	NA*
3	84	411	332	19.2	28.5
4	80	330	320	3.1	15.1
5	85	502	513	-2.2	25.1
6	89	428	408	4.7	17.7
7	93	541	486	10.2	26.8
**Mean**	**84**	**431**	**396**	**8.2**	**22.8**
SEM	2	26	29	2.7	2.0

The metabolic power while wearing the exoskeleton is compared to the metabolic power while wearing no device. The net metabolic power is the measured metabolic rate of walking minus the measured metabolic rate of standing. The average mechanical power is estimated by the applied voltage, motor velocity and experimentally determined actuator efficiency. The means and standard error mean (SEM) are presented in the bottom row. *The wireless telemetry malfunctioned for subject 2.

The measured electrical power suggests that the current exoskeleton can provide assistance for over 10 km of walking. The average electrical power was measured to be 49 ± 5.3 W. If one hundred percent of the battery’s energy was used, or 432 kJ, then the exoskeleton would have a battery life of 2.4 hours, or 13 km at 1.5 m/s.

### Augmentation factor comparison

**Figure 4:**
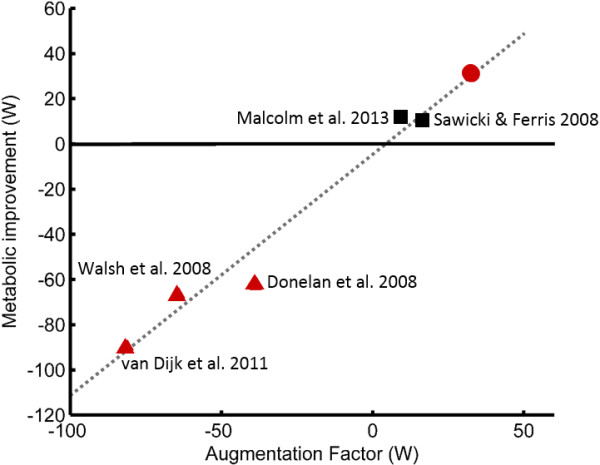
**Augmentation Factor (AF).** The AF was calculated for six devices and compared to the measured metabolic impact for each device [9,10,12,20,22]. Triangle markers are previously published autonomous devices, square markers are previously published tethered devices, and the circle marker is the presented autonomous exoskeleton of this study. The equation estimated by linear regression is *y* = 1.1*x* – 4 with an R^2^ equal to 0.98. In the AF equation, the *p*^*+*^ term is calculated by taking the positive work done by an exoskeleton during the gait cycle and dividing by the gait cycle duration. If the net-work done by the exoskeleton is negative, then *p*^*dis*^ is equal to this negative net-work divided by the gait cycle duration, otherwise, *p*^*dis*^ is zero.

To elucidate the mechanisms that underlie the metabolic performance of leg exoskeletons in walking, the AF was calculated for the presented device, as well as for other previously published exoskeletons that reported metabolic and mechanical power information (Figure 4 and Table 3). The included previous studies span a large range of device designs and metabolic impacts [9,10,12,20,22]. Researchers have used springs, damping elements, and actuators in various arrangements, with all previously published autonomous exoskeletons increasing metabolic energy consumption, and only non-autonomous, tethered exoskeletons decreasing metabolic demand. Using linear regression, the AF was able to explain the metabolic results of all exoskeletons with an R^2^ equal to 0.98.

**Table 3:** Augmentation factor calculation for six studies

**Study**	**Mass**	**p**^**+**^	**p**^**dis**^	**m**_**foot**_	**m**_**shank**_	**m**_**thigh**_	**m**_**waist**_	$\overset{4}{\underset{i=1}{\sum }}\beta_{i}m_{i}$	**AF**	**Metabolic impact**
	**(kg)**	**(W)**	**(W)**	**(kg)**	**(kg)**	**(kg)**	**(kg)**			
								**(W)**	**(W)**	**(W)**
van Dijk et al. [12]	75	3	0	3*	3*	3*	3*	88	-81	-90
Walsh et al. [9]	76	7	-5	1.41	1.41	5.12	3.66	69	-64	-67
Donelan et al. [10]	78	0	-9	0	1.6	1.6	0	18	-40	-62
Malcolm et al. [22]	66	9	0	0.4**	1.1**	0	0	12	10	12
Sawicki & Ferris [20,34]	80	16	0	.75	2.0	0	0	22	17	10
Present Study	84	23	0	0.5	1.75	0	1.71	23	33	36

Six studies were found that reported both metabolic results and applied mechanical power [9,10,12,20,22,35]. Powers that were not explicitly stated in literature were computed from provided graphs. The included exoskeletal masses are those worn on the foot, shank, thigh and waist (enumerated by *i* in equation 4). The *β* coefficient was 14.8 W/kg for mass added to the foot, 5.62 W/kg for the shank, 5.55 W/kg for the thigh, and 3.33 for the waist [17]. *The location of the exoskeleton mass was not described, so the mass was evenly distributed across the leg. **The exact location of the exoskeleton mass was not described, but the device is similar to the device studied by Sawicki & Ferris, so the same mass distribution was used.

## Discussion

The metabolic and mechanical power results support the hypothesis that a metabolic reduction during walking with a load can be achieved by an autonomous leg exoskeleton capable of providing substantial positive mechanical power with minimal added distal mass. The metabolic reduction provided by the exoskeleton is equivalent to reducing the payload by approximately 7 kg or 30% of the original payload of 23 kg [32].

The lightweight architecture of the exoskeleton allowed it to add positive mechanical power to the user without restricting the natural motions of the ankle joint. In order to apply a large torque about the ankle joint, it must be reacted by both a structure connected to the foot and a structure connected to the shank. Applying large forces to the body must be done carefully, in order to prevent discomfort or pain. One solution is to add a mechanical bearing in parallel with the ankle joint which greatly reduces the shear forces on the foot and shank [4,18,36]. However, the mechanical bearing must be closely aligned with the biological joint in order to prevent undesired forces and translations, and a bearing at the ankle joint adds non-trivial distal mass. Other exoskeletons have developed various methods to apply moments about a joint without a parallel bearing, but these devices have been limited in the amount of torque they can apply [37,38]. Instead, the presented device creates a rigid extension of the foot in only the plantar-flexion direction. Actuating the struts with a cord does not constrain the movement of the ankle and subtalor joints, and also reduces the distal mass, since the boot is only connected to the heel cord and a fiberglass strut. The freedom of the ankle and subtalor joints may have also contributed to the success of this device, but this effect would be difficult to measure and quantify.

The AF suggests the importance of an exoskeleton to provide substantial positive mechanical power to the wearer with minimal added leg mass and net-negative power dissipation. That is, using the fundamental characteristics of device mechanical power and mass, the potential metabolic impact can be estimated using the AF. The metabolic results observed in this study were achieved by a device with an AF of 33 W. Previously, the most successful published exoskeleton (Malcom et al.) had an AF of 10 W. That device was tethered, having an external power supply and yet showed one third the metabolic improvement (12 W) of the device we present in this paper (36 W) [22]. Historically, previously published autonomous exoskeletons have not provided a metabolic improvement, demonstrated by their negative AF values (Figure 4). In accordance with the AF, to design a non-dissipating exoskeleton (*p*^*dis*^ *= 0*) that reduces the metabolic cost of walking, it must apply a mean positive power, *p*^*+*^ > *η* ∑ *β*_*i*_*m*_*i*_ (4). Furthermore, a purely dissipative device (*p*^*+*^ *= 0 and p*^*dis*^ *< 0*) will have a negative AF (*e.g.*[10]) and an estimated increase in metabolic cost. Future investigators can use the AF to estimate the metabolic impact of an exoskeletal leg design, reducing the likelihood of the device unintentionally increasing walking metabolic energy once fabricated.

Certain assumptions are made when calculating the AF that must be considered carefully. The AF does not account for different control schemes and only accounts for mechanical power and added distal mass. That is, the AF assumes that power is added in a biomimetic fashion where positive and negative power are added by the exoskeleton during phases of the gait cycle when the joint is also applying positive or negative power, respectively. Current and previous exoskeletal control methodologies apply power in this way. The muscle-tendon efficiency of 0.41 used by the AF is also an estimate based on two studies [20,22]. The muscle-tendon efficiency may also be specific for each joint and activity [34,39]. More studies are needed to precisely determine how this apparent muscle-tendon efficiency is affected by joint and activity. Furthermore, the transfer of mechanical energy between joints is not fully considered when calculating the AF. Literature suggests that energy is transferred between joints via bi-articular muscle-tendon action when one joint is performing negative power and another is exhibiting positive power [26,27]. This may explain why the device presented by Donelan et al. increased the metabolic rate higher than predicted by the AF [10]; a knee exoskeleton that harvests electrical energy from negative phases of knee mechanical work may reduce the mechanical energy transferred to the hip via bi-articular muscle-tendon units, potentially increasing muscular hip work and adversely affecting metabolism. The possible metabolic advantage of an exoskeleton reducing the negative mechanical power applied by muscle-tendon units is also not considered by the AF. Providing a joint with negative power during phases of eccentric muscle work may reduce the metabolic burden. However, muscles are substantially more efficient at eccentric work than they are at concentric work [8], thus, aiding the joints during eccentric phases is less effective at reducing the metabolic expenditure during walking, when compared to aiding concentric contraction phases.

The equation found by linear regression of the AF and measured metabolic effect, *y* = 1.1*x*-4, (95% C.I. slope: [0.86, 1.33] (p = 0.0002), intercept: [-15, 7] (p = 0.38)), highlights some important features of the AF. When no device is worn the AF should predict no metabolic change; that is, a device that does not supply power and has no mass should have no metabolic effect. The nearly zero y-intercept correctly accounts for this case. The slope of the linear regression is approximately one, indicating the ability of the AF to predict metabolic change caused by a worn exoskeleton. The AF includes the aforementioned assumptions as well as the empirically-estimated muscle-tendon efficiency for positive mechanical work (*η*). Future work will focus on the validation of these assumptions.

In this work, comparisons are made between exoskeletons that were tested with load carriage and those that were tested during unloaded conditions. The effect of added load on the metabolic improvement of an exoskeleton is unknown, but may be an advantage compared to unloaded studies. Because of the limited number of studies documenting the metabolic impact of exoskeletal walking, the calculation of the AF in this study assumed that the muscle-tendon efficiency was not affected by different loading conditions.

Future improvements of the autonomous exoskeleton should increase the versatility and controllability of the device. Reducing the posterior protrusion of the struts will allow for a greater range of motion. An integrated force sensor will also allow for more precise torque control. While using motor state to apply a torque impulse was successful, more sophisticated control paradigms will require a greater level of force measurement. The maximum applied mechanical power and relative timing varied by subject due to the subtle differences in how the subjects utilized the exoskeleton. The timing of the push-off assistance was statically determined before the exoskeleton trial and did not adapt with changes in step period, due to wearing the exoskeleton. This provided a consistent timing that the subjects could rely on, but it also may have forced the subjects to walk at a non-optimal cadence while wearing the exoskeleton. Future control strategies should be able to adapt to the natural cadence of the user. Finally, a fully autonomous exoskeleton presents the opportunity to develop controls for activities other than walking in a laboratory. Specifically, gait transitions and terrain adaptations will be an exciting area of future investigation.

## Conclusions

In this study, a fully autonomous leg exoskeleton was described that reduced the metabolic burden of walking during load carriage. In accordance with the Augmentation Factor framework, the applied mechanical power characteristics of the device coupled with the distal mass were critical factors to its success. The presented exoskeleton could reduce the metabolic burden of individuals expected to carry substantial loads. Instead of reducing the metabolic burden, the device may allow them to carry greater loads at their nominal metabolic cost. A lightweight autonomous powered exoskeleton enables the investigation of real-world applications including the complex controls needed for various terrains and conditions outside of the laboratory.

## Competing interests

A patent provisional has been filed with the U.S. Patent Office describing the exoskeletal mechanism documented in this manuscript.

## Authors’ contributions

LM conceived of the exoskeleton architecture, designed the exoskeleton, performed data collection and analysis, and drafted sections of the manuscript. ER assisted in the development of the device, participated in data collection and analysis, and drafted sections of the manuscript. HH conceived of the study, assisted in the exoskeleton design and assisted in drafting the manuscript. All authors read and approved the final manuscript.

## Supplementary Material

Additional file 1 Movie 1– Subject walking with autonomous exoskeleton. Click here for file
